# Supplementary material for: A high-throughput small molecule screen identifies farrerol as a potentiator of CRISPR/Cas9-mediated genome editing
Source: eLife. 2020 Jul 9;9:e56008. doi: 10.7554/eLife.56008 (PMC7380943; doi:10.7554/eLife.56008)
Supplement: Transparent reporting form [file elife-56008-transrepform.docx]

***eLife’s* transparent reporting form**

We encourage authors to provide detailed information *within their submission* to facilitate the interpretation and replication of experiments. Authors can upload supporting documentation to indicate the use of appropriate reporting guidelines for health-related research (see [EQUATOR Network](http://www.equator-network.org/%20)), life science research (see the [BioSharing Information Resource](https://biosharing.org/)), or the [ARRIVE guidelines](http://www.plosbiology.org/article/info:doi/10.1371/journal.pbio.1000412) for reporting work involving animal research. Where applicable, authors should refer to any relevant reporting standards documents in this form.

If you have any questions, please consult our Journal Policies and/or contact us: [editorial@elifesciences.org](mailto:editorial@elifesciences.org).

**Sample-size estimation**

- You should state whether an appropriate sample size was computed when the study was being designed
- You should state the statistical method of sample size computation and any required assumptions
- If no explicit power analysis was used, you should describe how you decided what sample (replicate) size (number) to use

Please outline where this information can be found within the submission (e.g., sections or figure legends), or explain why this information doesn’t apply to your submission:

The sample size used in our study was decided by following previous studies focusing on similar scientific questions, such as (Yao, et al. 2017) and (Li, et al. 2017). All detailed information on sample size is indicated in related figure or figure legends.

**Replicates**

- You should report how often each experiment was performed
- You should include a definition of biological versus technical replication
- The data obtained should be provided and sufficient information should be provided to indicate the number of independent biological and/or technical replicates
- If you encountered any outliers, you should describe how these were handled
- Criteria for exclusion/inclusion of data should be clearly stated
- High-throughput sequence data should be uploaded before submission, with a private link for reviewers provided (these are available from both GEO and ArrayExpress)

Please outline where this information can be found within the submission (e.g., sections or figure legends), or explain why this information doesn’t apply to your submission:

Drug screening, analysis on repair efficiency, EdU assay and cell cycle were repeated for three times. At least 150 cells from three independent experiments were included for analysis of comet assay. Foci number of at least 50 cells from three independent experiments were counted for analyzing the recruitment of repair factors. t-test was employed for further statistical analysis in these assays.

For knock-in analysis by FACS, the experiments were repeated for three times. For knock-in efficiency analysis by either fluorescent imaging or genotyping, the experiments were repeated for at least three times and the ‘N’ is indicated in related figure or figure legends. 𝛘^2^-test was employed for statistical analysis in these assays.

We do not generate any high-throughput sequencing data in this work.

**Statistical reporting**

- Statistical analysis methods should be described and justified
- Raw data should be presented in figures whenever informative to do so (typically when N per group is less than 10)
- For each experiment, you should identify the statistical tests used, exact values of N, definitions of center, methods of multiple test correction, and dispersion and precision measures (e.g., mean, median, SD, SEM, confidence intervals; and, for the major substantive results, a measure of effect size (e.g., Pearson's r, Cohen's d)
- Report exact p-values wherever possible alongside the summary statistics and 95% confidence intervals. These should be reported for all key questions and not only when the p-value is less than 0.05.

Please outline where this information can be found within the submission (e.g., sections or figure legends), or explain why this information doesn’t apply to your submission:

In this work, mean, Standard Deviation (s.d.) and Standard Error of the Mean (s.e.m.) are indicated immediately after the numerical results. The statistical method of each result is indicated in the figure legends.

(For large datasets, or papers with a very large number of statistical tests, you may upload a single table file with tests, Ns, etc., with reference to sections in the manuscript.)

**Group allocation**

- Indicate how samples were allocated into experimental groups (in the case of clinical studies, please specify allocation to treatment method); if randomization was used, please also state if restricted randomization was applied
- Indicate if masking was used during group allocation, data collection and/or data analysis

Please outline where this information can be found within the submission (e.g., sections or figure legends), or explain why this information doesn’t apply to your submission:

**Additional data files (“source data”)**

- We encourage you to upload relevant additional data files, such as numerical data that are represented as a graph in a figure, or as a summary table
- Where provided, these should be in the most useful format, and they can be uploaded as “Source data” files linked to a main figure or table
- Include model definition files including the full list of parameters used
- Include code used for data analysis (e.g., R, MatLab)
- Avoid stating that data files are “available upon request”

Please indicate the figures or tables for which source data files have been provided:

A total of 722 small molecules were screened for their influence on HR and NHEJ repair efficiency (shown as a dot plot in Figure 1G), and the detailed list of these molecules and their effect on repair efficiency are provided as the “Figure-source data-1” file.

All data generated or analyzed during this study are included in the manuscript and supporting files. Source data files have been provided for Figure 1, 2, 3, 4, 5, 6 and figure supplements contained within “Source data files”. Primer sequences named “Table 1-source data 1” are also included as “Source data files”.
